# Atomic-level organization of vicinal acid–base pairs through the chemisorption of aniline and derivatives onto mesoporous SBA15†
†Electronic supplementary information (ESI) available. See DOI: 10.1039/c6sc01229a


**DOI:** 10.1039/c6sc01229a

**Published:** 2016-06-09

**Authors:** Bilel Hamzaoui, Anissa Bendjeriou-Sedjerari, Eva Pump, Edy Abou-Hamad, Rachid Sougrat, Andrei Gurinov, Kuo-Wei Huang, David Gajan, Anne Lesage, Lyndon Emsley, Jean-Marie Basset

**Affiliations:** a King Abdullah University of Science and Technology (KAUST) , KAUST Catalysis Center (KCC) , Thuwal , 23955-6900 , Saudi Arabia . Email: jeanmarie.basset@kaust.edu.sa; b Université de Lyon , Institut de Sciences Analytiques (CNRS/ENS-Lyon/UCB-Lyon 1) , Centre de RMN à Très Hauts Champs , 69100 Villeurbanne , France; c Institut des Sciences et Ingénierie Chimiques , Ecole Polytechnique Fédérale de Lausanne (EPFL) , CH-1015 Lausanne , Switzerland

## Abstract

The design of novel heterogeneous catalysts with multiple adjacent functionalities is of high interest to heterogeneous catalysis. Herein, we report a method to obtain a majority of bifunctional acid–base pairs on SBA15. Aniline reacts with SBA15 by opening siloxane bridges leading to *N*-phenylsilanamine–silanol pairs. In contrast with ammonia treated surfaces, the material is stable under air/moisture. Advanced solid state MAS NMR (2D ^1^H–^1^H double-quantum, ^1^H–^13^C HETCOR) experiments and dynamic nuclear polarization enhanced ^29^Si and ^15^N spectra demonstrate both the close proximity between the two moieties and the formation of a covalent Si–N surface bond and confirm the design of vicinal acid–base pairs. This approach was successfully applied to the design of a series of aniline derivatives of bifunctional SBA15. A correlation between the substituent effects on the aromatic ring (Hammett parameters) with the kinetics of a model Knoevenagel reaction is observed.

## Abstract 2

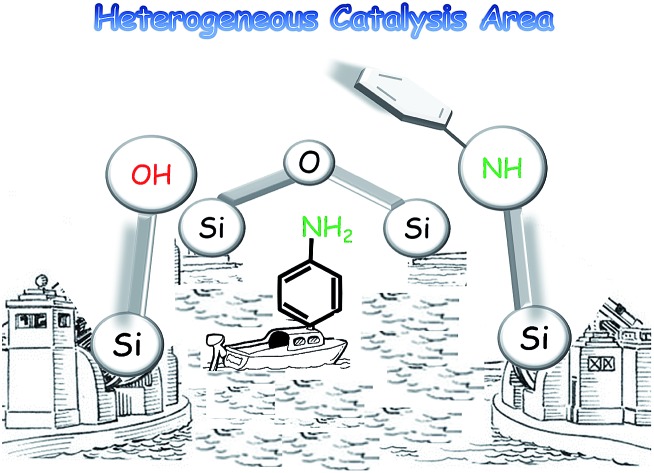
The design of novel heterogeneous catalysts with multiple adjacent functionalities is of high interest to heterogeneous catalysis.

## Introduction

One of the major current challenges in heterogeneous catalysis is the ability to develop multifunctional catalyst systems where each active site plays a distinct role in the overall catalytic process (cascade approach). To date, two main approaches to introduce functionalities into mesoporous materials exist: the soft templating strategy for the synthesis of organic–inorganic hybrid materials^1^–^6^ and the surface organometallic chemistry (SOMC) methodology for the generation of well-defined surface species.^7^–^11^

In the soft templating method, the inorganic materials provide the surface area and the porosity. The organic active site, linked to the surface *via* an alkyl spacer, can be randomly distributed or organized.^12^–^21^ However, the resulting materials are composed of complex mixtures of surface species statistically and randomly spread on the surface which are consequently difficult to characterize. Accordingly, the structure of their active site is generally not known at the molecular level.

In the SOMC methodology,^7^ the generation of well-defined surface species is achieved by understanding the reaction of organometallic complexes with the inorganic materials which act as a rigid ligand. This approach presents the advantage of establishing a structure–activity relationship, and provides molecular-level insight for the design and prediction of new catalysts for new reactions.^22^,^23^ Indeed, SOMC has been successful in designing “multifunctional” single site catalysts that are able to perform alkane metathesis *via* a multistep mechanism. However, the surface–complex bond is usually a σ-bonded oxygen ligand, *e.g.*, siloxy [(≡Si–O–)ML_*n*_], with M = metal and L = ligands, in the primary coordination sphere and the requirement for oxygen limits the development of SOMC methods. It would then be highly desirable to tune the coordination sphere of the metal center by designing surface ligands in close proximity to the surface to preserve the rigidity of the ensemble “surface ligand/complex”. By tuning the electronic and/or steric properties of the surface ligand, new catalysts and new reactions will be discovered.

**Scheme 1:**
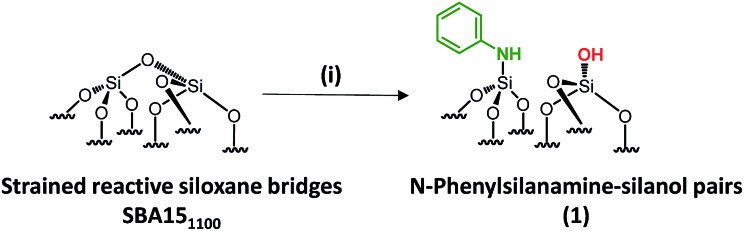
Synthesis of paired *N*-phenylsilanamine/silanols **1***via* the chemisorption of dry aniline on SBA15_1100_ in toluene at 80 °C for 20 h.

In 2013, our group developed a new strategy to create an N-donor surface pincer ligand where the functional groups are well-organized into silylamine and silanol pairs on mesoporous SBA15 materials, named [N,O]SBA15. This was achieved by opening strained siloxane bridges at 200 °C *via* treatment with ammonia, by analogy with the ring opening of epoxides by ammonia.^24^–^26^ By reaction with an organometallic complex (zirconium tetraneopentyl), the expected bipodal(siloxy-)(amido-)zirconium bis neopentyl was obtained.^27^–^29^ However, the successful formation of [≡Si–NH_2_][≡Si–OH] surface groups is associated with experimental, economical and safety disadvantages, such as the need for a high flow rate (200 mL min^–1^) of pure, expensive and corrosive ammonia, and the resulting materials being moisture sensitive. Aside from that, further chemical modifications of [N,O]SBA15 to offer opportunities to provide tunable steric and electronic properties were impossible without affecting the structural parameters of the materials. To face these issues, we investigate an alternative approach based on the chemisorption of dry aniline onto highly dehydroxylated SBA15 (1100 °C), which has never been reported (Scheme 1). The resulting new materials are inexpensive, easy to prepare and moisture stable and their performance as acid–base catalysts is evaluated in the Knoevenagel condensation where a controllable distance between two antagonist functionalities is known to be an influential factor in the enhancement of the reactivity.^12^,^14^,^19^,^30^–^35^

## Results and discussion

**Fig. 1:**
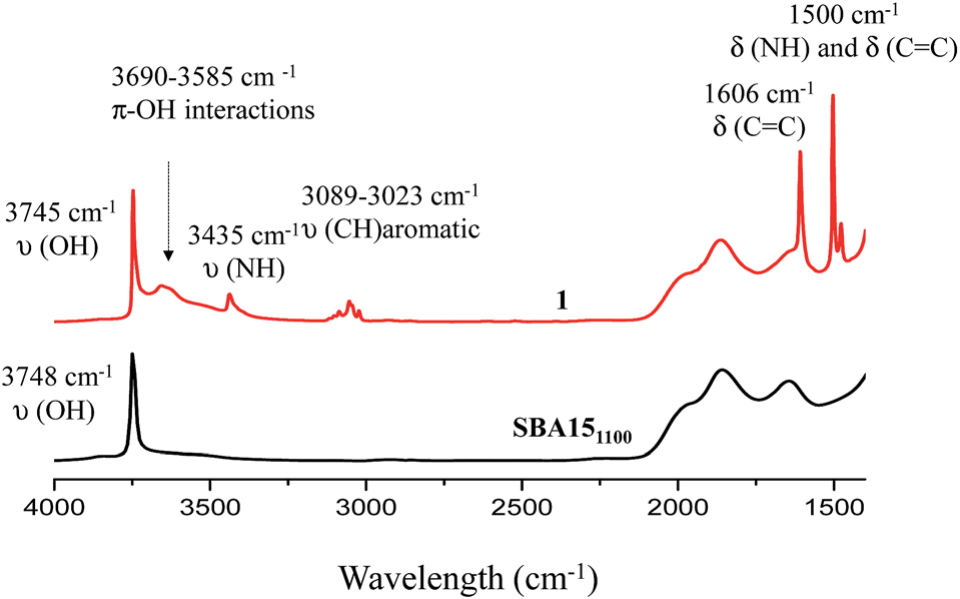
FT-IR spectra of SBA15_1100_ (black) and **1** (red).

Well-ordered hexagonal mesoporous silica, SBA15, was chosen as a support because of its high thermal stability (up to 1200 °C), its high surface area (700 m^2^ g^–1^) and its large and uniform pore diameter (6 nm) and relatively thick walls (3 to 6 nm).^36^ Thermal treatment at 1100 °C under vacuum (10^–5^ mbar) yields the condensation of adjacent silanols and generates a support that contains a surface of mainly strained siloxanes along with a small amount of isolated silanols (<0.4 OH per nm^2^).^37^–^39^ As described in Scheme 1, the reaction of SBA15_1100_ with dry aniline was performed in a solution in toluene at 80 °C for 20 h. The resulting material **1** was evacuated at room temperature overnight under high vacuum (10^–5^ mbar) and characterized by FT-IR spectroscopy. Comparison of the FT-IR spectra of SBA15_1100_ and **1** (Fig. 1) reveals a slight increase in intensity of the characteristic [*ν*_s_(OH)] band with a red shift from 3748 to 3745 cm^–1^. Additionally, the typical single sharp infrared bands characteristic of secondary amines,^40^ here *N*-phenylsilylamine appears at 3435 and 1500 cm^–1^. They correspond to the [*ν*(NH)] and [*δ*(NH)], respectively. Vibrational bands of the aromatic group are clearly visible at 3089–3023 cm^–1^ [*ν*(CH)], at 1606 and 1500 cm^–1^ [*δ*(C=C)] (overlap a NH band). Finally, the shoulder in the range of 3690–3585 cm^–1^ is assigned to electronic interactions of the π system of the aromatic group with the newly formed adjacent silanol (π–OH interactions).^41^,^42^ It is important to mention that no reversible adsorption aniline (physisorbed) remains on the support after the reaction. Indeed, the adsorption of aniline, which we do not see, would lead to a decrease of the isolated silanol band due to the hydrogen bonding of aniline to surface silanol, and the appearance of two bands at 3395 and 3300 cm^–1^ assigned to the N–H stretching vibration of physisorbed aniline.^41^,^42^

**Fig. 2:**
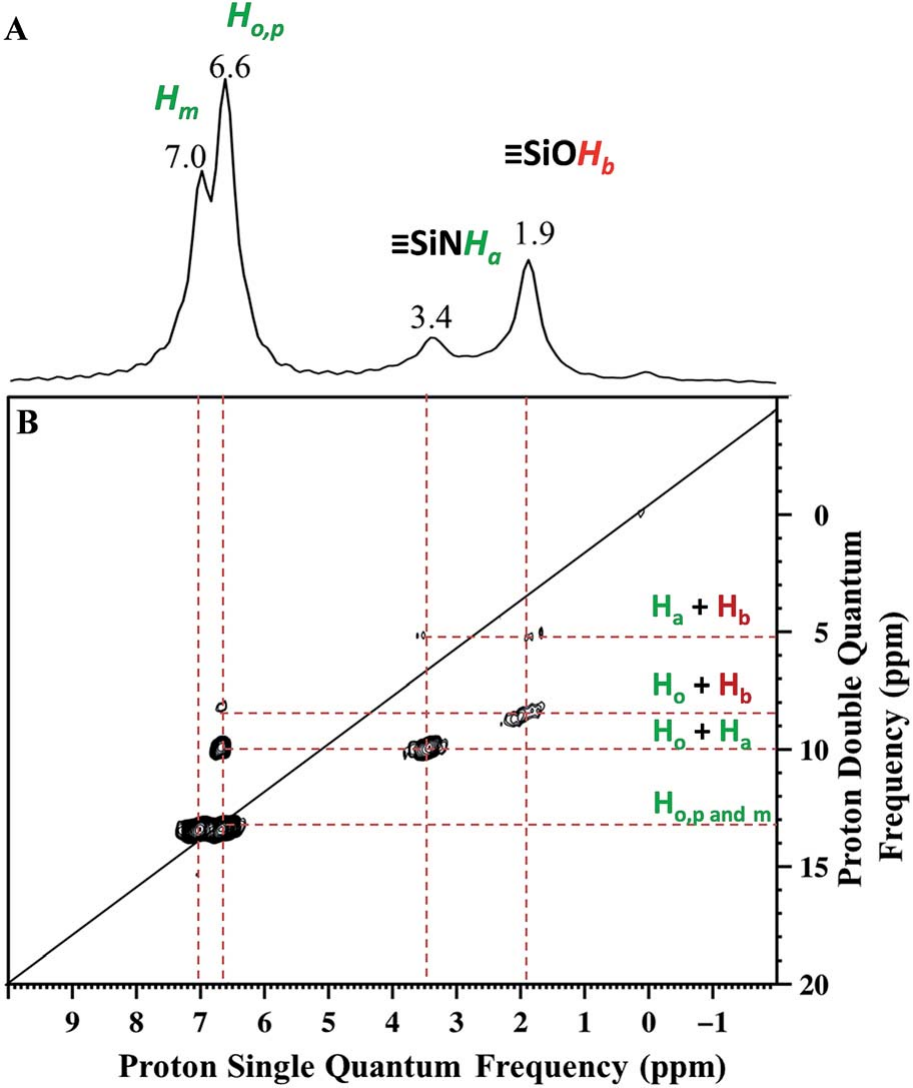
(A) ^1^H MAS NMR spectrum of **1**. (B) DQ rotor-synchronized 2D ^1^H MAS NMR spectrum of **1** (see ESI† for details).

Solid state NMR was used to characterize the pairwise nature of the atomic-level organization of the supported organic functionalities, *N*-phenylsilanamine and silanol. The ^1^H magic-angle-spinning (MAS) spectrum (Fig. 2A) shows four clear resonances at 1.9, 3.4, 6.6 and 7 ppm. The chemical shift at 1.9 ppm is assigned to the ≡SiO***H*** proton. Its value appears slightly downfield compared to the chemical shift of ≡SiOH (1.7 ppm) generated by treatment with ammonia.^27^ This shift might be due to the proximity of the protons of the aromatic ring which induces π–OH interactions. The chemical shift at 3.4 ppm is attributed to the proton of the ≡Si–N***H***–Ph.^43^

The two intense signals at 6.6 ppm correspond to the protons of the aromatic ring in *ortho* (***H***_***o***_) and *para* (***H***_***p***_) positions. The proton in the *meta* position (***H***_***m***_) appears at 7 ppm (Fig. 2A). To confirm these proton assignments, the synthesis of a model molecular silsesquioxane bearing a *N*-phenylsilanamine group was carried out (see ESI, Fig. S1†). The ^1^H liquid-state NMR spectrum of the resulting silsesquioxane is in agreement with the ^1^H MAS spectrum of **1** and confirms the formation of a σ bond between the nitrogen of aniline and the SBA15 surface.

Further information about the presence of vicinal functionalities are obtained from the 2D ^1^H–^1^H double quantum (DQ) NMR spectrum (Fig. 2). The 2D DQ spectrum of **1** shows a weak correlation between the proton resonance of ≡SiO***H*** at 1.9 ppm and the proton resonance of ≡SiN***H***Ph at 3.4 ppm [5.3 ppm in F1: *δ*_H_(OH) + *δ*_H_(NH) = 1.9 + 3.4] (Scheme 2.1), indicating that the two protons are in close proximity, typically <5 Å.

**Scheme 2:**
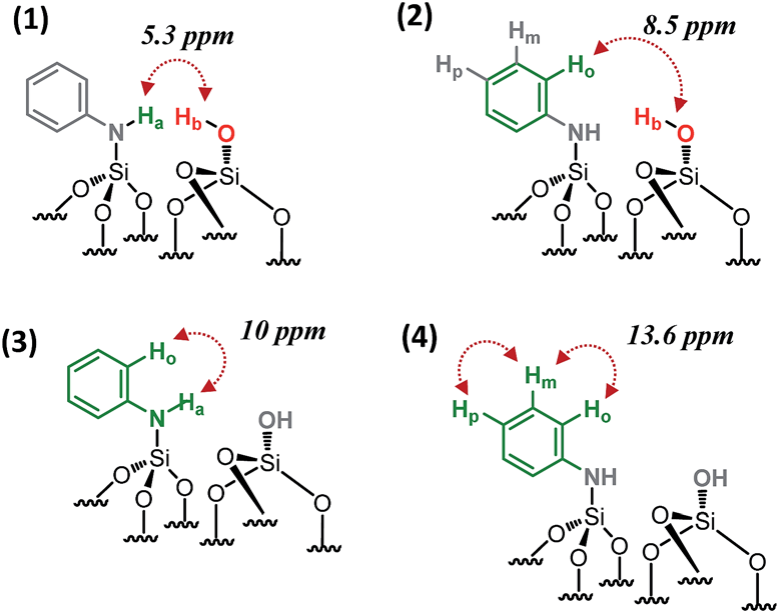
Schematic showing the observed proximities of the paired *N*-phenylsilanamine/silanol groups in **1** from the 2D ^1^H–^1^H DQ NMR spectrum.

Moreover, the paired organization is reinforced by the presence of a correlation between the proton resonance of ≡SiO***H*** and the aromatic proton resonances of the neighboring ≡SiN***H***Ph at 6.6 ppm [8.5 ppm in F1: *δ*_H_(OH) + *δ*_H_(***H***_***o***_) = 1.9 + 6.6] (Scheme 2.2). The correlation most likely arises from *ortho* position ***H***_***o***_. Note that ***H***_***m***_ does not correlate with the silanol indicating that the aromatic ring is oriented in such a way that ***H***_***o***_ is closest to the silanol. An additional correlation is observed between the proton resonance of ≡SiN***H***Ph at 3.4 ppm and the proton of the aromatic ring in *ortho* position ***H***_***o***_ at 6.6 ppm [10 ppm in F1: *δ*_H_(OH) + *δ*_H_(***H***_***o***_) = 3.4 + 6.6] (Scheme 2.3). Finally, strong correlation peaks are observed between all the protons of the aromatic ring at 13.6 ppm (Scheme 2.4).

Interestingly, the fact that no correlations between two silanols [≡SiO***H***] [≡SiO***H***] (1.9 × 2 = 3.8 ppm in F1) as well as between two [≡SiN***H***Ph] [≡SiN***H***Ph] (2 × 3.4 = 6.8 ppm in F1) are observed clearly demonstrates that the majority of sites are isolated “acid–base” pairs [≡SiN***H***Ph]: [≡SiO***H***].

We conclude that dry aniline reacts with strained siloxane bridges to generate vicinal *N*-phenylsilanamine and silanol groups. These results are consistent with those obtained with FT-IR spectroscopy.

In the ^13^C CP-MAS NMR spectrum (Fig. S4A†), the signals of the aromatic ring appear clearly at 118, 120, 129 and 143 ppm corresponding to the ***C***_***o***_H, ***C***_***p***_H, ***C***_***m***_H and to the quaternary carbon linked to the nitrogen surface ≡Si–NH–***C***^***IV***^, respectively. These assignments are in accordance with those of the silsesquioxane model (see ESI, Fig. S2†). The ^1^H–^13^C HETCOR spectrum demonstrates a strong correlation between these four carbon resonances and the proton resonances at 6.6 and 7 ppm attributed to the proton of aromatic group (see ESI, Fig. S4B†). These results confirm that the integrity of the organic fragment is maintained under the reaction conditions.

To identify the formation of a covalent bond between the silica surface and the organic fragment, ≡ ***SiN***HPh, ^29^Si and ^15^N solid state NMR spectra are required, but are not practical using conventional methods due to their low sensitivity at natural isotopic abundance.

**Fig. 3:**
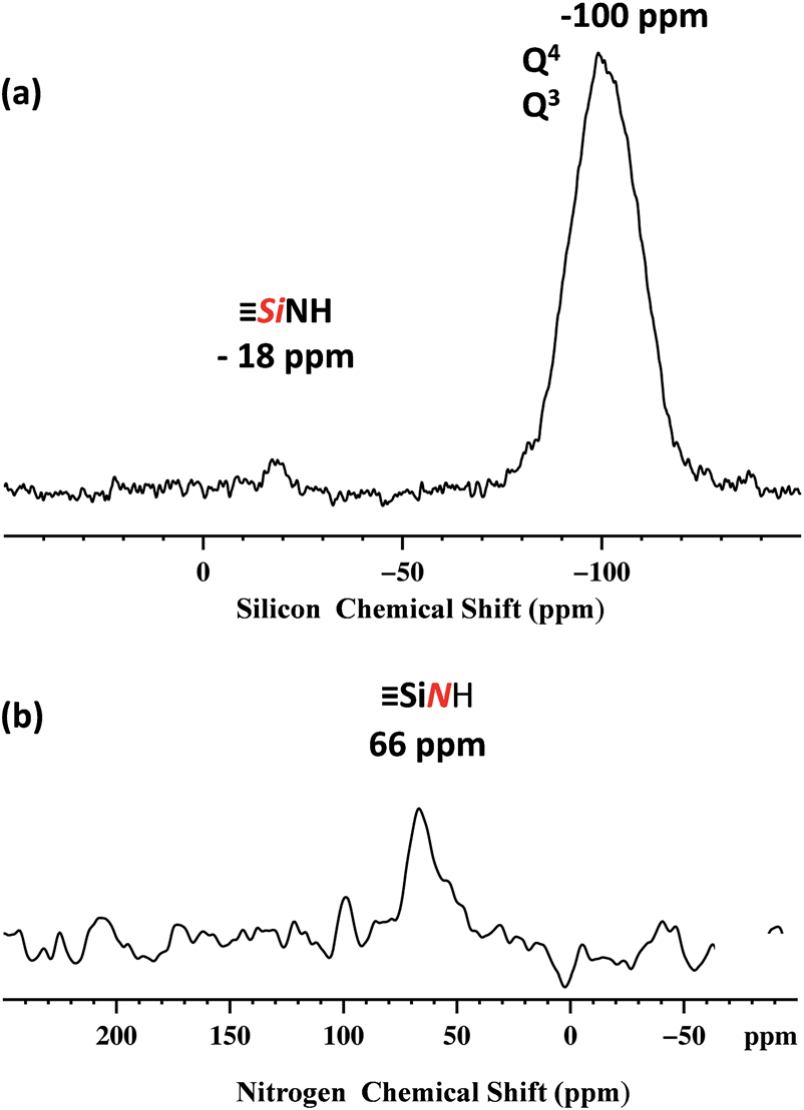
400 MHz DNP SENS spectra of **1** (20 mg) impregnated with a 16 mM solution of TEKPOL^53^ in 1,1,2,2-tetrachloroethane at 8 kHz MAS frequency with a sample temperature of 100 K. (a) ^29^Si DNP enhanced CP/MAS with a CP contact time of 5 ms, a 3 s polarization delay and 1024 scans. Exponential line broadening of 60 Hz was applied prior to Fourier transformation. (b) ^15^N DNP enhanced CP/MAS with 5 ms CP contact time, a 3 polarization delay and 16 000 scans. Exponential line broadening of 150 Hz was applied prior to Fourier transformation. In both (a) and (b) for comparison, spectra are shown with both for both μwave on and off.

Dynamic nuclear polarization surface enhanced NMR (DNP SENS)^44^,^45^ has recently been introduced and demonstrated to overcome these difficulties for the characterization of hybrid materials, and was used here.^46^–^49^ In this work, DNP yielded *ε*_H_ ∼ 256 (defined as the ratio of signal intensities of spectra acquired with and without microwave irradiation, Fig. S5, ESI†). The natural abundance ^29^Si DNP SENS spectrum of **1** (Fig. 3a) displays a signal centered at –100 ppm (intense) and a signal at –18 ppm (weak). According to the literature the former is attributed to both [(≡ ***Si***O)_3_***Si***OE], with E = Si or H (commonly dubbed Q^4^ and Q^3^)^50^,^51^ and the latter is assigned to the [≡ ***Si***NHPh].^52^

The natural abundance ^15^N DNP SENS spectrum shows a single peak at 66 ppm (Fig. 3b) and it is in agreement with the ^15^N liquid-state NMR spectrum of the model molecular silsesquioxane, for which the ^15^N chemical shift appears at 62 ppm (Fig. S3, ESI†).

Textural characterization is used to evaluate the preservation of the mesoporous materials, here by nitrogen sorption porosimetry, small angle X-ray diffraction (XRD), and Transmission Electronic Microscopy (TEM). The small angle X-ray diffraction patterns of **1** (Fig. S6, ESI†) exhibit three clear peaks (*d*_100_, *d*_110_ and *d*_200_) in the 2*θ* range of 0.7–4°. They confirm the presence of a well-ordered hexagonal mesophase with a *d*_100_ spacing of 86.28 Å (see ESI, Table S1†). The structure of the mesoporous materials is thus maintained throughout the chemisorption of dry aniline.

Analyses of the nitrogen adsorption/desorption isotherms yielded BET surface areas of **1** of approximately 512 m^2^ g^–1^ (*versus* 679 m^2^ g^–1^ for SBA_1100_) and pore volumes of 0.65 cm^3^ g^–1^ (*versus* 0.9 cm^3^ g^–1^ for SBA_1100_). Also, **1** showed type IV isotherms (Fig. S7, ESI†), with clear H1-type hysteresis loops associated with capillary condensation in the mesopores and with regular pore sizes of 50 Å. The textural parameters of sample **1** are summarized in Table S1† and are characteristic of mesoporous materials. The surface coverage *α* of the organic moieties based on the carbon content was calculated as described by Jaroniec *et al.*^54^ Using eqn (S1) (see ESI†), a carbon content of 1.42 wt% was determined for **1**, which translates into a surface coverage of 0.3 μmol m^–2^. The low surface coverage supports the results of N_2_ sorption experiments and indicates the functionalization of the SBA15_1100_.

**Fig. 4:**
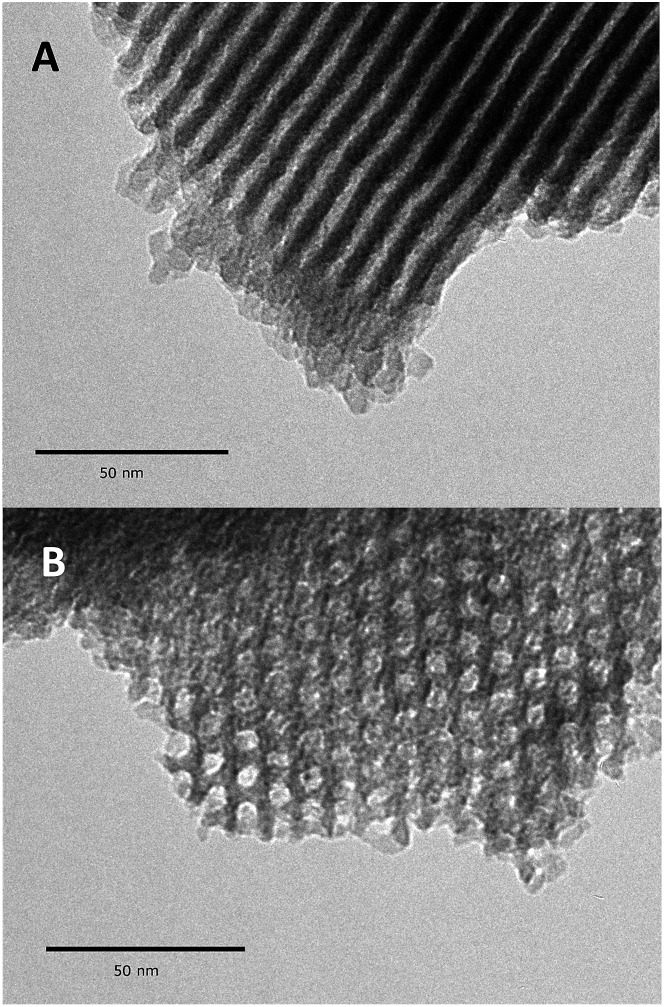
Transmission electron micrographs of **1** at different tilt angles: in the direction perpendicular to the pore axis (B), and in the direction of the mesopores axis (B).

Further evidence for a well-ordered hexagonal mesostructure is provided by the TEM images (Fig. 4), which are representative of mesoporous SBA15. After the high thermal treatment (1100 °C, 10^–5^ mbar) and the dissociative chemisorption of aniline (80 °C, toluene, 20 h), the mesoporous structure is still regular over the whole particle of **1**.

**Scheme 3:**
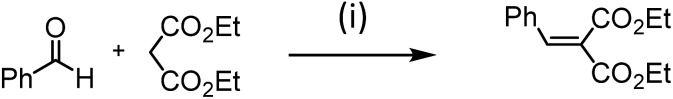
Knoevenagel condensation between benzaldehyde and diethylmalonate. (i) The reaction was performed in sealed flask in which each reactant (2 mmol) and the catalyst (20 mg) were added to dry ethanol (5 mL) and the reaction mixture was refluxed at 80 °C for 24 h.

A materials with atomic organization of acid–base pairs should exhibit cooperative catalytic behavior for the Knoevenagel condensation of benzaldehyde with diethyl malonate (p*K*_a_ = 13) (Scheme 3).^55^–^57^ The Knoevenagel condensation between a carbonyl group and activated methylene compounds is one of the most useful C=C bond forming reactions. It produces several important key intermediates such as α, β unsaturated products widely used for the synthesis of therapeutic drugs, functional polymers and fine chemicals.^58^,^59^ The Knoevenagel reaction is the right catalytic reaction as it is considered as the model reaction to evaluate the basic strength of bifunctional acid/base materials.^55^–^57^

In the literature, several studies have revealed an efficient catalysis by cooperative acid–base pairs well organized on mesoporous silica.^16^,^60^ Those previous studies have been supported by recent work from Jones *et al.*^61^ where the catalytic activity of amino-propyl functionalized MCM-41 decreases drastically when the ≡Si–OH are capped with trimethylsilyl group (≡Si–OSiMe_3_). So, weakly acidic silanols play a vital role in the cooperative catalytic cycle as well as the spatial organisation of the acid–base functionalities.^60^ In the mechanism, activation of the carbonyl group occurs on weak Brönsted acid sites and the basic sites extract the proton from methylene (Scheme S1, ESI†). In this case, the control of the distance is a key parameter to enhance the reactivity of the Knoevenagel condensation.^12^,^14^

**Scheme 4:**
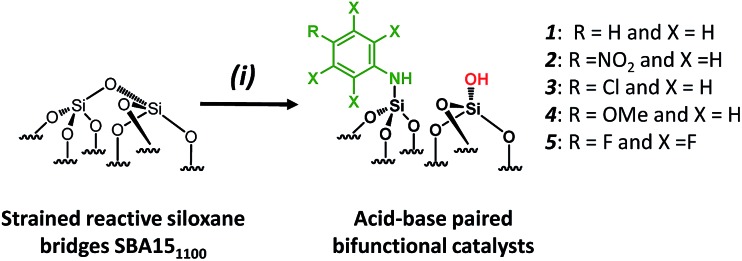
Synthesis of acid–base paired catalysts *via* the chemisorption of dry aniline derivatives on SBA15_1100_ in toluene at 80 °C for 20 h.

For comparison purposes, a series of bifunctional mesoporous materials with different electronic properties were successfully synthesized through the same approach (Scheme 4). All the materials were characterized by FT-IR and ^1^H-MAS solid state NMR spectroscopy (Fig. S8 and S9†).

All the FT-IR spectra of catalysts **2–5** display the characteristic vibration bands of *ν*(OH) (3745 cm^–1^), *ν*(NH) and *δ*(NH), 3435 and 1500 cm^–1^ respectively. Vibrational bands of the aromatic group are still present at 3089–3023 cm^–1^ [*ν*(CH)], at 1606 and 1500 cm^–1^ [*δ*(C=C)] (overlapping a NH band). The shoulder characteristic of the electronic interactions of the π system of the aromatic group with the newly formed adjacent silanol (π–OH interactions) in the range of 3690–3585 cm^–1^ is again observed for all catalysts (weak in the case of catalyst **5**).

All the ^1^H NMR spectra feature the characteristic signal of ≡SiOH and ≡SiNH at around 2 ppm and 3.5–3.9 ppm, respectively. As expected, the protons in *ortho* and *meta* position to electron donating (OMe) and electron withdrawing (Cl, NO_2_) substituents show distinct upfield and downfield shifts (Fig. S9†).

Their catalytic performance were tested (Table 1, entry 1–5) and all the samples showed good catalytic ability. Entry 1 showed higher activity than entry 2. The nitro group is a strongly electron-withdrawing group (EWG) and thus, catalyst (**2**) is a weaker base than catalyst (**1**). A chloro group in the *para* position is a slightly EWG, so catalyst (**3**) exhibits better activity than (**2**) and is slightly less active than (**1**). Introducing an electron-donating group (EDG) as a *p*-methoxy group in the catalyst (**4**) enhances the catalytic performance in the Knoevenagel reaction. Among all these catalysts, (**4**) exhibits the best performance whereas (**5**) exhibits the lowest due to the base weakening effect.

**Table 1:** Catalytic activity of acid–base paired catalysts **1–7** for the Knoevenagel condensation between benzaldehyde and diethyl malonate

Entry	Catalyst	N loading^*a*^ (mmol g^–1^)	Yield^*b*^ (%)	TON^*c*^
1	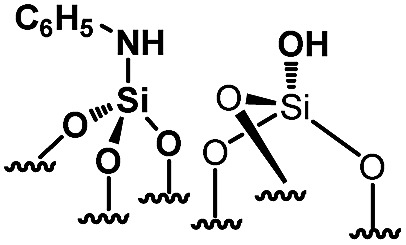	0.23	59	257
2	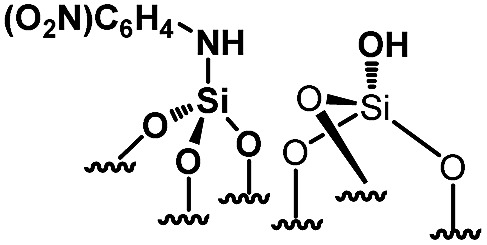	0.22	35	159
3	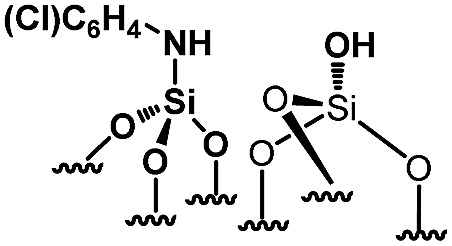	0.18	43	238
4	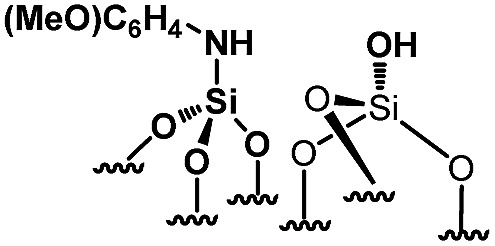	0.21	64	304
5	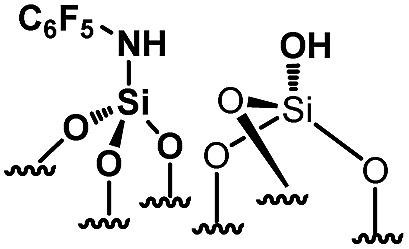	0.18	24	133
6	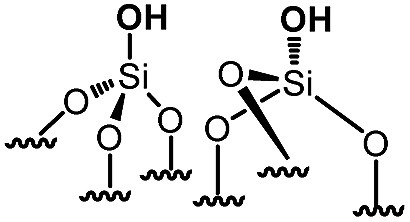	0	0	0
7	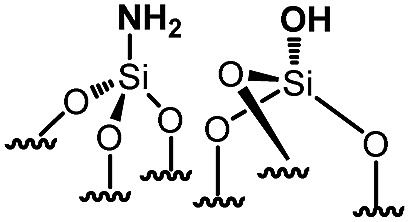	0.35	18	51

^*a*^Determined by elemental analysis.

^*b*^Determined by GC analysis after 24 h.

^*c*^Turnover number (TON) = number of moles of product per number of moles of active amine site.

Besides this, the catalytic results of this series of acid–base paired catalysts (Table 1, entry 1–5) were compared to two other materials (Table 1, entry 6 and 7): an unmodified SBA15 displaying different silanols (vicinal, geminal) (**6**), [N,O]SBA15 where primary amine and silanol groups are proximal (**7**).^27^ As expected (**6**) shows no activity as no basic sites are present. (**7**) contains primary amines which are supposed to be the strongest base; yet it gives only 18% conversion after 24 h. (**1**) and (**4**) yield better conversion although their basicity is lower than that of (**7**). These results are explained by the higher stability of these catalysts under the experimental conditions (ethanol is the solvent and water is produced during the Knoevenagel reaction). Indeed, the ≡SiNH_2_ group is well-known to be easily hydrolyzed.^32^,^34^

In parallel, the stability of (**7**) and (**1**) towards ethanol was monitored by FT-IR spectroscopy. After 5 min in contact with ethanol, the FT-IR spectrum (Fig. S10, ESI†) of (**7**) shows complete disappearance of the characteristic bands of the ≡SiNH_2_ group [*ν*_s_(NH_2_) = 3535, *ν*_s_(NH_2_) = 3445 and *δ*(NH_2_) = 1550 cm^–1^]. However the FT-IR spectrum of (**1**) shows the characteristic bands of ≡Si–NHPh, [*ν*(NH) = 3435 cm^–1^] even after 1 h in contact with dry ethanol. In addition, during the catalytic test with catalyst **1**, no leaching of aniline was detected by both GC-FID and GC-MD (Fig. S11, ESI†).

## Conclusions

The opening siloxane bridges approach was successfully established to create an atomic organization of well-defined bi-functional acid–base pairs on mesoporous SBA15. This approach is based on an analogy between organic epoxides and strained siloxanes (≡Si–O–Si≡) of mesoporous SBA15_1100_. The generation of well-defined adjacent *N*-phenylsilanamine–silanol pairs was unambiguously determined through FTIR, 2D solid state NMR, XRD, nitrogen sorption and TEM. This way to design bi-functionalized mesoporous surface offers new opportunities to modify the electronic and steric properties of mesoporous silica useful for heterogeneous catalysis.

## Supplementary Material

Supplementary information
