# Supplementary material for: Atomic-level organization of vicinal acid–base pairs through the chemisorption of aniline and derivatives onto mesoporous SBA15
Source: Chem Sci. 2016 Jun 9;7(9):6099–105. doi: 10.1039/c6sc01229a (PMC6024177; doi:10.1039/c6sc01229a)
Supplement: Supplementary file 1 [file SC-007-C6SC01229A-s001.pdf]

Electronic Supplementary Material (ESI) for Chemical Science.
This journal is © The Royal Society of Chemistry 2016

Electronic Supporting Information

Atomic-Level Organization of Vicinal Acid-Base Pairs through the Chemisorption of Aniline and Derivatives onto Mesoporous SBA15

Bilel Hamzaoui,† [a] Anissa Bendjeriou-Sedjerari,†[a] Eva Pump,[a] Edy Abou-Hamad,[a] Rachid Sougrat,[a] Andrei Gurinov,[a] Kuo-Wei Huang,[a] David Gajan,[b] Anne Lesage,[b] Lyndon Emsley[c] and Jean-Marie Basset.*[a]

[a] KAUST Catalysis Center (KCC), King Abdullah University of Science and Technology, Thuwal-23955-6900, Saudi Arabia,

[b] Université de Lyon, Institut de Sciences Analytiques (CNRS / ENS-Lyon / UCB-Lyon 1), Centre de RMN à Très Hauts Champs, 69100 Villeurbanne, France.

[c] Institut des Sciences et Ingénierie Chimiques, Ecole Polytechnique Fédérale de Lausanne (EPFL), CH-1015 Lausanne, Switzerland

All experiments were carried out under controlled atmosphere. Treatments of the surface species were carried out using high vacuum lines (1.34 Pa) and glove box techniques. Toluene was distilled on Na-benzophenone and degassed through freeze pump thaw cycles.

Infrared spectra were recorded on a Nicolet 6700 FT-IR spectrometer equipped with a cell under controlled atmosphere.

Elemental analyses were performed at Mikroanalytisches Labor Pascher (Germany).

Nitrogen adsorption–desorption isotherms at 77 K were measured using a Micromeritics ASAP 2024 physisorption analyzer. Specific surface areas were calculated following typical BET procedures. Pore size distribution was obtained using BJH pore analysis applied to the desorption branch of the nitrogen adsorption/desorption isotherm.

The small-angle X-ray powder diffraction (XRD) data were acquired on a Bruker D8 advance diffractometer using Cu Kα monochromatic radiation (λ=1.054184 Å) to confirm the hexagonal ordered structure of the samples.

One-dimensional ^{1}H MAS and ^{13}C CP/MAS solid state NMR spectra were recorded on Bruker AVANCE III spectrometers operating at 400 MHz resonance frequencies for ^{1}H. Experiments at 400 MHz employed a conventional double-resonance 3.2 mm CP/MAS probe. In all cases the samples were packed into rotors under an inert atmosphere inside gloveboxes. Dry nitrogen gas was utilized for sample spinning to prevent degradation of the samples. NMR chemical shifts are reported with respect to the external references TMS and adamantane. For ^{13}C CP/MAS NMR experiments, the following sequence was used: 90° pulse on the proton (pulse length 2.4 s), then a cross-polarization step with a contact time of typically 2 ms, and finally acquisition of the ^{13}C NMR signal under high-power proton decoupling. The delay between the scans was set to 5 s to allow the complete relaxation of the 1H nuclei, and the number of scans ranged between 3000 and 5000 for ^{13}C and was 32 for ^{1}H. An exponential apodization function corresponding to a line broadening of 80 Hz was applied prior to Fourier transformation.

The 2D ^{1}H–^{13}C heteronuclear correlation (HETCOR) solid state NMR spectroscopy experiments were conducted on a Bruker AVANCE III spectrometer using a 3.2 mm MAS probe. The experiments were performed according to the following scheme: 90° proton pulse, t1 evolution period, CP to ^{13}C, and detection of the ^{13}C magnetization under TPPM decoupling. For the cross-polarization step, a ramped radio frequency (RF) field centered at 75 kHz was applied to the protons, while the ^{13}C channel RF field was matched to obtain an optimal signal. A total of 32 t1 increments with 2000 scans each were collected. The sample spinning frequency was 8.5 kHz. Using a short contact time (0.5 ms) for the CP step, the polarization transfer in the dipolar correlation experiment was verified to be selective for the first coordination, to lead to correlations only between pairs of attached 1H–13C spins (C–H directly bonded).

^{1}H–^{1}H Multiple-Quantum Spectroscopy. Two-dimensional double-quantum (DQ) and triple-quantum (TQ) experiments were recorded on a Bruker AVANCE III spectrometer operating at 600 MHz with a conventional double-resonance 3.2 mm CP/MAS probe, according to the following general scheme: excitation of DQ coherences, t1 evolution, z filter, and detection. The spectra were recorded in a rotor synchronized fashion in t1 by setting the t1 increment equal to one rotor period. One cycle of the standard back-to-back (BABA) recoupling sequences was used for the excitation and reconversion period. Quadrature detection in w1 was achieved using the States-TPPI method. An MAS frequency of 22 kHz was used. The 90° proton pulse length was 2.5 μs, while a recycle delay of 5 s was used. A total of 128 t1 increments with 32 scans per each increment were recorded. The DQ frequency in the w1 dimension corresponds to the sum of two single-quantum (SQ) frequencies of the two coupled protons and correlates in the w2 dimension with the two corresponding proton resonances.

DNP-solid state NMR. DNP experiments were performed on a 400 MHz (^{1}H/electron Larmor frequencies) Bruker Avance III solid-state NMR spectrometer equipped with a 263 GHz gyrotron. The sweep coil of the main super conducting coil was set so that microwave irradiation occurred at the positive enhancement maximum of TEKPOL. At low temperature double resonance 3.2 mm probe configured for ^{29}Si and after for ^{15}N CP/MAS. Sample temperature during DNP experiments were around 100K. DNP enhancements were measured by comparing the intensity of spectra acquired with and without continuous wave irradiation. A 20 mg of powdered material was impregnated with 20 μL of 16mM of TEKPOL tetrachloethane solution. Impregnated materials were then packed into sapphire rotors inside glovebox. For the CPMAS experiments the following

sequence was used: 900 pulse on the proton, then cross-polarization with a contact time of 5 ms and finally acquisition of the ^{29}Si and ^{15}N signal under high power decoupling. SPINAL-64 heteronuclear ^{1}H decoupling was employed during acquisition.

Transmission Electron Microscopy (TEM). Sample **1** was spread on a copper grid precoated with a holey carbon layer. Imaging was performed on a Tecnai Biotwin transmission electron microscope (FEI Company) operating at 120 kV. Data was acquired at 1um defocus and recorded on a Eagle CCD camera 4kx4k (FEI company)

Preparation of 1 (impregnation method). In a schlenk, $SBA15_{1100\ C}$ (0.5 g) was reacted with dry aniline (2.5 ml) in toluene for 24 h at 80°C. After filtration and four washing cycles, the resulting white powder was dried for 8 hr under dynamic vacuum (< 10^{-5}mbar).

Elemental analysis (mass. %). C (1.42) and N (0.35) corresponding to C/N ratio of 5.1

Preparation of N-phenylsilylanamine silsesquioxane SQ-3

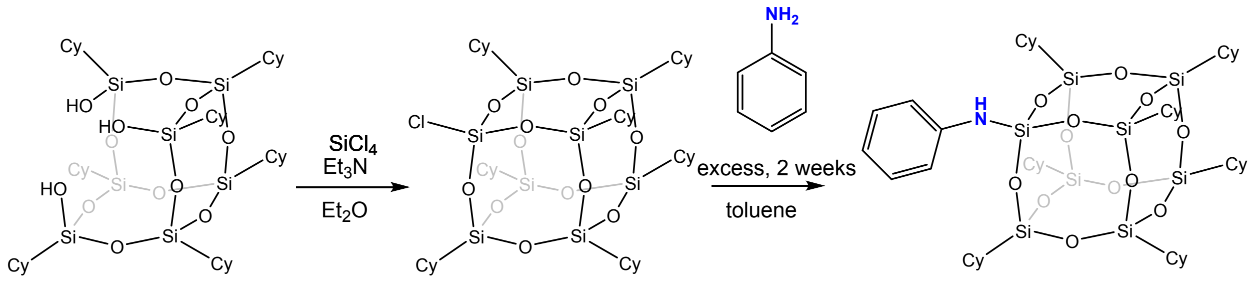

SQ - 1 **SQ - 2** **SQ - 3**

1,3,5,7,9,11,13 - Heptacyclohexyltricyclo [7.3.3.15,11] heptasiloxane - 3,7,13 - triol **SQ-1** (1.0 g, 0.92 mmol, 1.0 eq.) was reacted with $SiCl_4$ (0.13 mL, 1.1 mmol, 1.2 eq.) in the presence of NEt_3 (0.40 mL, 2.9 mmol, 3.1 eq.) in dry Et_2O (50 mL) under inert conditions.
The reaction was stirred overnight at reflux. After cooling down, the precipitate was filtered and the solvent was removed. The white solid residue was washed with hot acetonitrile/toluene, 1/1 (v/v), filtered and dried to dryness to isolate 1,3,5,7,9,11,14 – heptacyclohexyltricyclo [7.3.3.15,11] octaasiloxane - 15 - chloride **SQ-2**.
Yield: 0.92 g (97 %) as white solid.
In a next step, toluene was added (20 mL) to react SQ-2 (0.92 g, 0.89 mmol, 1.0 eq.) with aniline (0.46 mL, 5.0 mmol, 5.7 eq.) in presence of NEt_3 (0.33 mL, 2.4 mmol, 2.7 eq.) under inert conditions. The reaction was stirred at room temperature for 18 days. Then, the white precipitate was filtered and the toluene was removed. To isolate 1,3,5,7,9,11,14 – heptacyclohexyltricyclo [7.3.3.15,11] octaasiloxyl – 15 - aniline **SQ-3**, column chromatography was performed in DCM (Rf = 0.98) to remove the remaining aniline and in ethylacetate/hexane, 1/1 (v/v) to remove the remaining impurities. The purity of **SQ-3** was followed by ^{1}H NMR (400 MHz, C_6D_6) and ^{13}C NMR (100 MHz, C_6D_6)

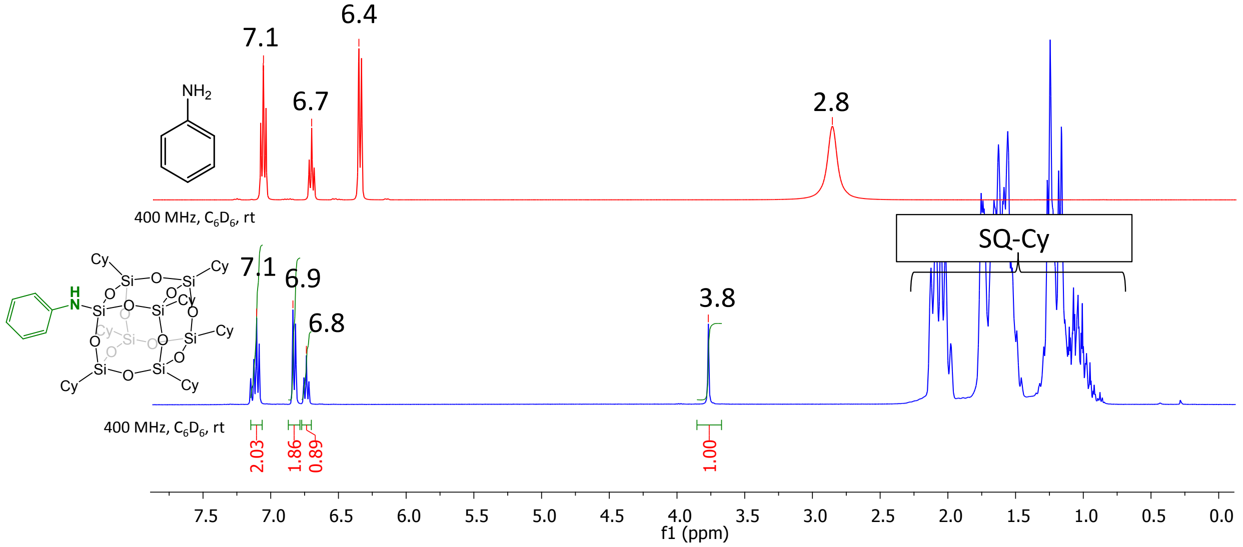

Figure S1: ¹H liquid NMR of aniline and **SQ-3** in C_6D_6

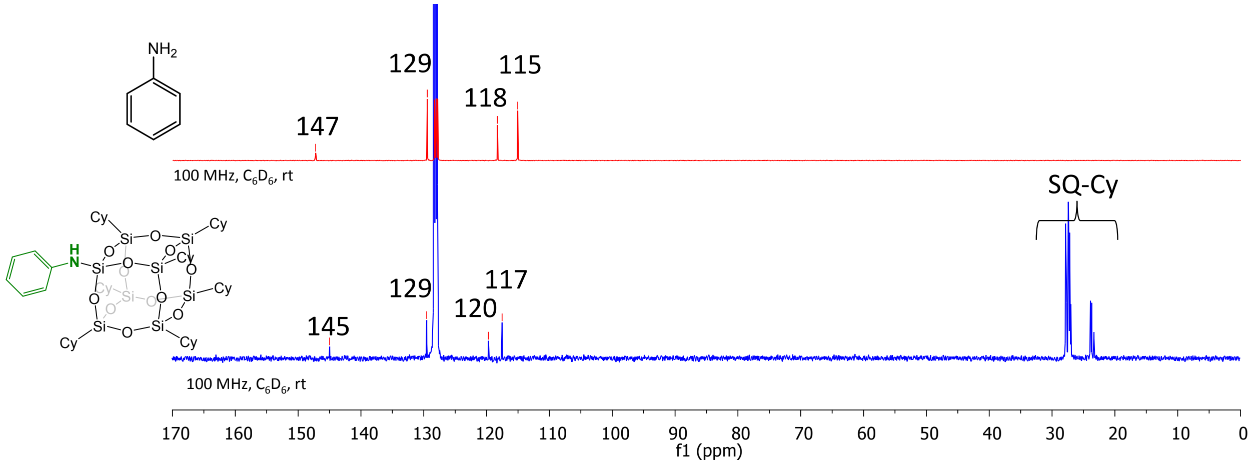

Figure S2: ¹³C liquid NMR of aniline and **SQ-3** in C_6D_6.

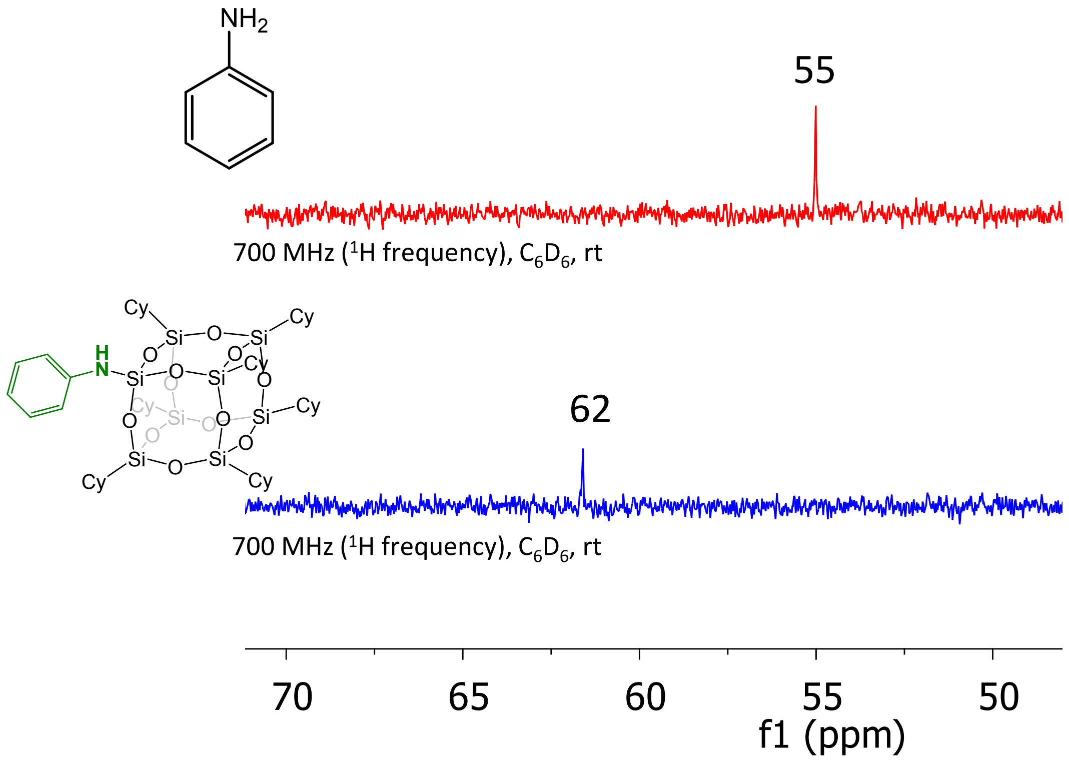

Figure S3: ^{15}N liquid NMR of aniline and **SQ-3** in C_6D_6.

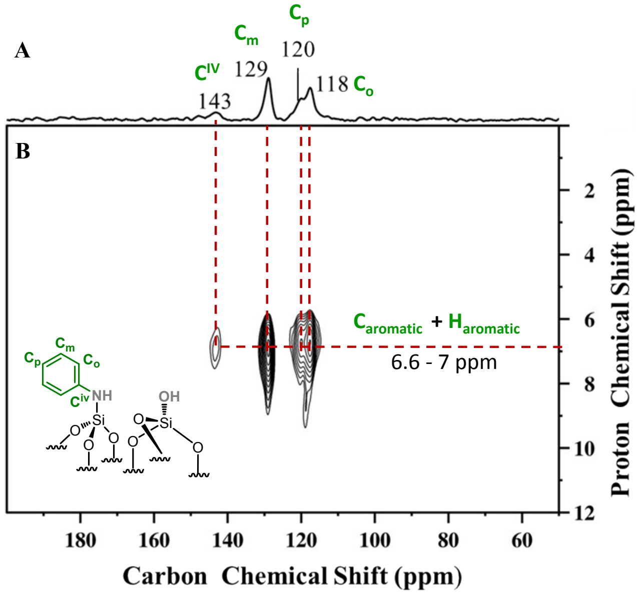

Figure S4: (A) ^{13}C CP-MAS NMR of 1. (B) 2D contour plot of the aromatic region of ^{1}H-^{13}C HETCOR spectrum of 1 (see ESI† for details).

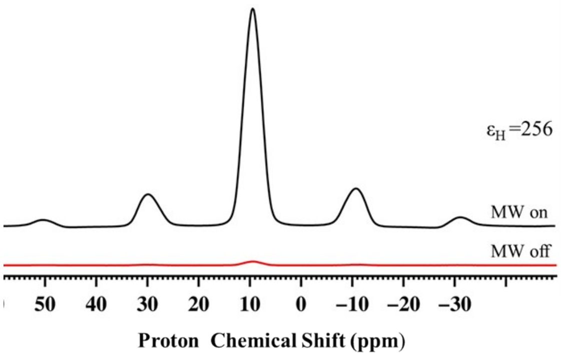

Figure S5: 400MHz DNP SENS spectra of **1** (20mg) impregnated with 16mM solution of TEKPOL in 1,1,2,2-tetrachloroethane with 8kHz MAS frequency. The microwave (MW) irradiation at 263 GHz was switched on or off continuously. ^{1}H MAS NMR were recorded using 16 scans for MW off and 4 scans for MW on and recycle delay of 3s .

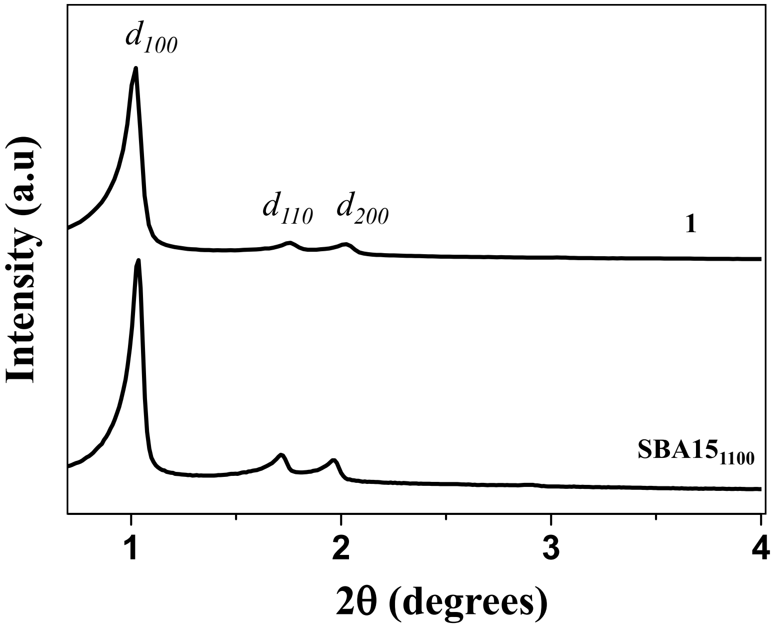

Figure S6: Small angle XRD patterns of SBA1100 and **1**

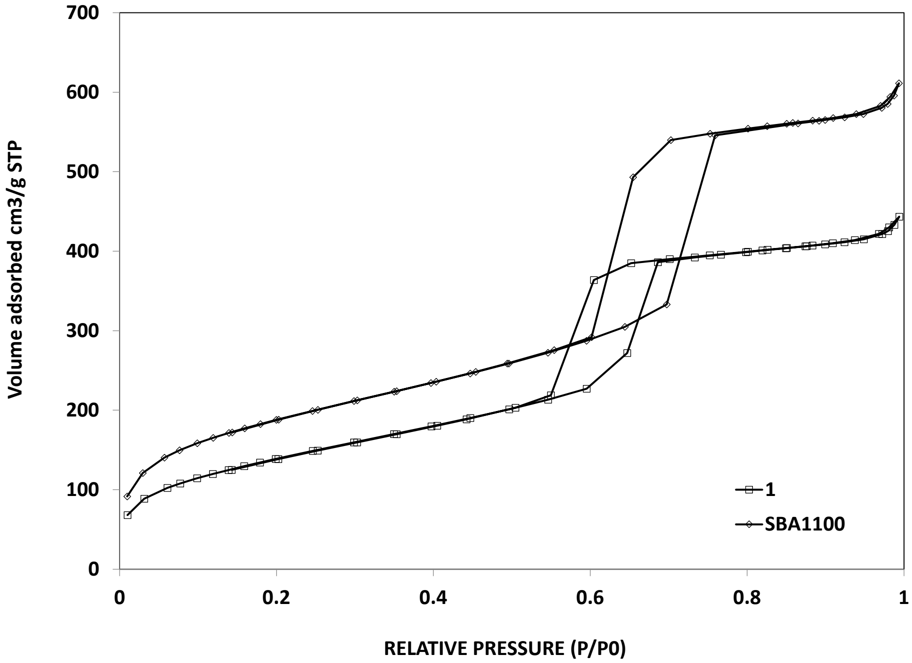

Figure S7. Nitrogen adsorption/desorption isotherm at 77 K of SBA_{1100} and **1.**

Table S1. Textural parameters of 1 from Nitrogen sorption combined with small angle X-ray diffraction

	d_{100}[a] (Å)	a_0[b] (Å)	Wall thickness[c] (Å)	V_p[d] ($cm^3.g^{-1}$)	D_p[e] (Å)	S_{BET} ($m^2.g^{-1}$)
**SBA_{1100}**	85.4	98.6	41.6	0.90	57	679
**1**	86.28	99.6	49	0.65	50	512

[a] d_{100} spacing.
[b] $a_0 = 2d_{100}/\sqrt{3}$, hexagonal lattice parameter from XRD.
[c] Calculated by a_0 – pore size.
[d] Total pore volume at P/P0 = 0.97. [e] Pore size from desorption branch applying the BJH pore analysis.
Note: The micropore volume corresponds to 0.2% of the total pore volume of the SBA_{1100} (on the basis of α_s plot). [1-2]

Determination of the surface coverage

The surface coverages (per m² of unmodified $SBA15_{1100}$) of N-phenylsilanamine ligands (α) bonded to the surface were estimated from the carbon contents of the materials using Equation (S1):

$$\alpha\ (mol/m^2) = \frac{P_C}{1200n_C - P_C(M-1)} \Big/ S_{BET}$$

Where, P_C = carbon percentage in the N-phenylsilanamine bonded phase; M are the molecular mass of the ligand attached, n_C are the number of carbon atoms per ligandS attached and S_{BET} is the BET specific surface are in m^2/g

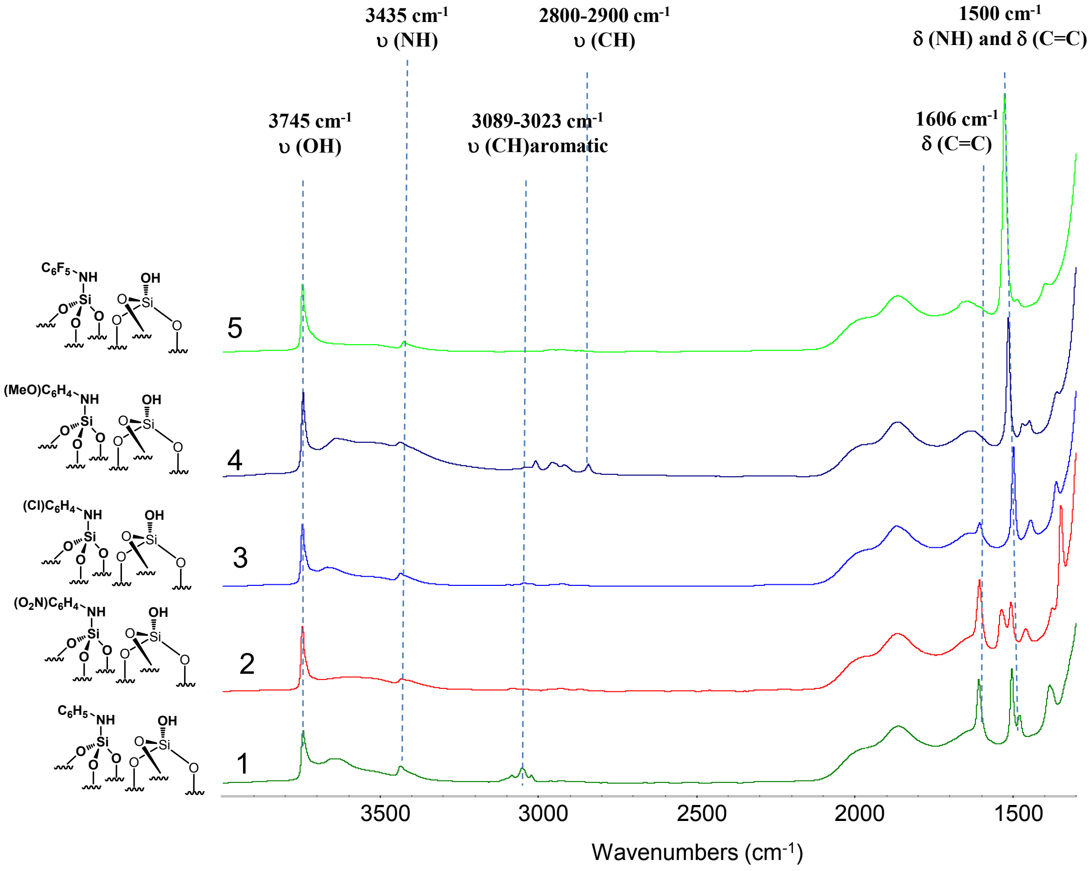

Figure S8. FT-IR spectra of materials 1-5

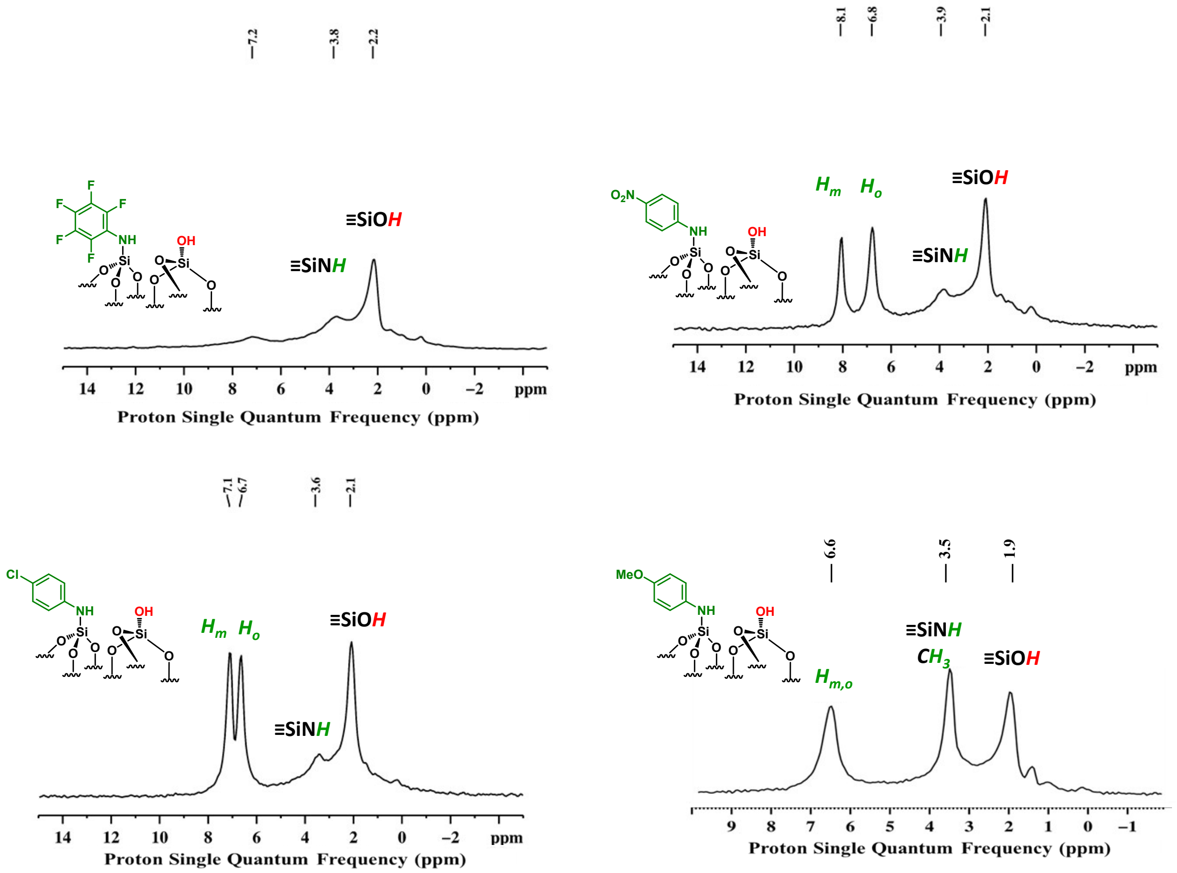

Figure S9. ^{1}H MAS solid state NMR of catalyst **2-5**

Catalytic performance for Knoevenagel reaction

In a typical experiment, the reactants were mixed in anhydrous ethanol and 0.02 g of the dried catalyst into the reactor and heated at 80 ºC. After the reaction, the catalyst was separated by filtration. The products were analyzed using a Chrompak CP 9000 gas chromatograph (GC) equipped with 30 m × 0.32 mm RTX-50 capillary column and FID detector. Individual reaction product was identified by GC-mass spectrometry (HP5971 mass spectrometer connected with a 30 × 0.25 mm RTX-50 capillary column)

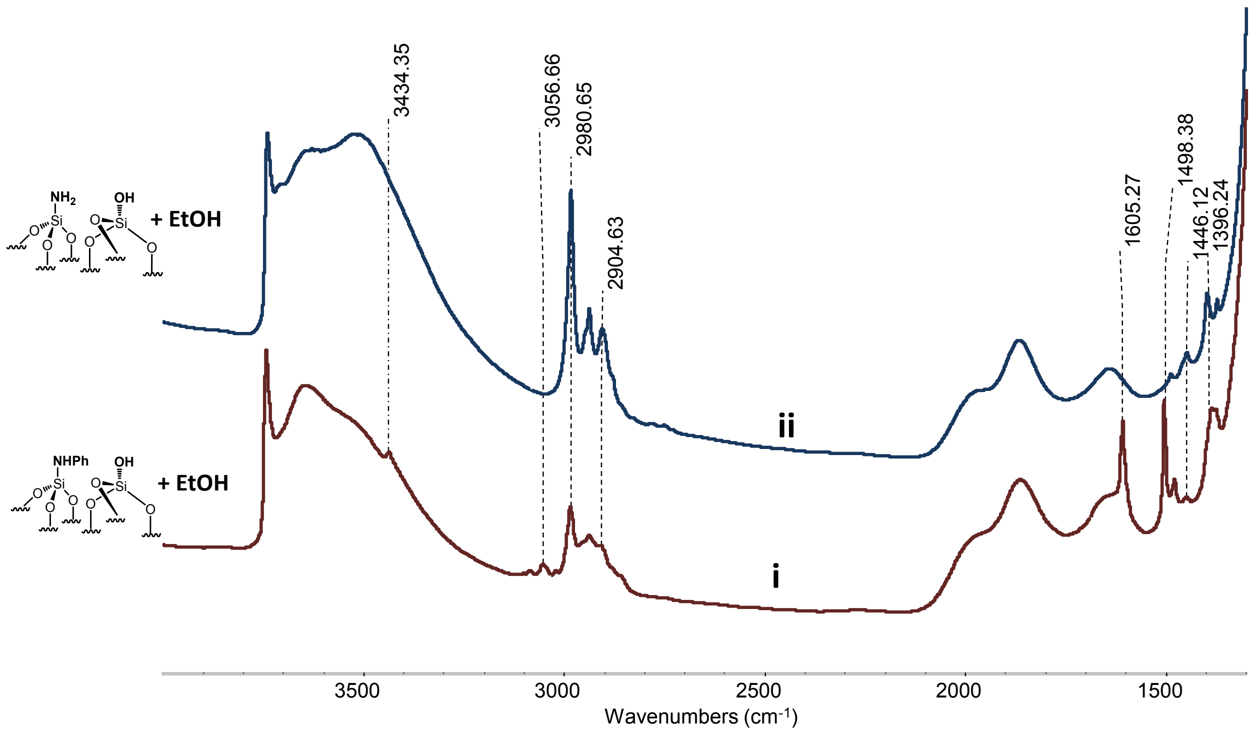

Figure S10. (i) FT-IR spectrum of [NHPh,O] SBA15, **1** after 1hr in contact with dry ethanol and followed by evacuation at 10^{-5} mbar, 100 ºC for 12h: the characteristic bands of ≡SiNHPh, [υ(NH) = 3435 cm^{-1}] is still observed. (ii) FT-IR spectrum of [N,O] SBA15, after 5 min in contact with dry ethanol and followed by evacuation at 10^{-5} mbar, 100 ºC for 12h: complete disappearance of the characteristic bands of ≡SiNH2 group [$\upsilon_s(NH_2)$ = 3535 , $\upsilon_{as}(NH_2)$ =3445 and $\delta(NH_2)$ = 1550 cm^{-1}].

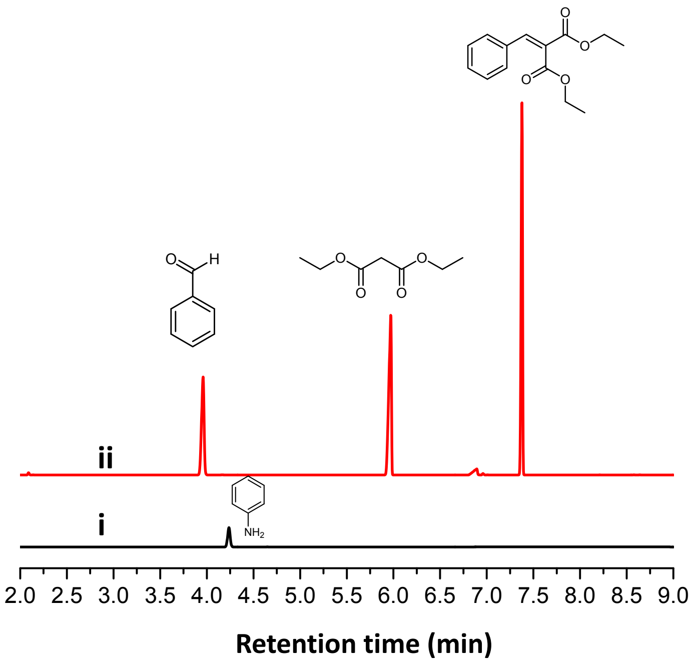

Figure S11. (i) GC-MD Chromatogram of pure dry aniline and GC chromatogram of reactant and product of Knoevenagel condensation.

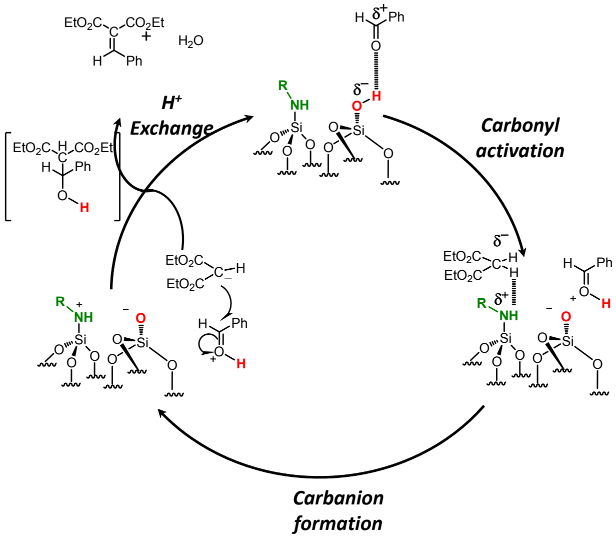

Scheme S1. A proposed mechanism of Knoevenagel condensation involving well-defined acid/base pairs bifunctional catalysts (with R = C_6H_5, $(NO_2)C_6H_4$, $(Cl)C_6H_4$, $(MeO)C_6H_4$, C_6F_5 and H.

Reference

[1] Jaroniec, M.; Kruk, M.; Olivier, J. P. *Langmuir* **1999,** *15*, 5410

[2] Ryong Ryoo and Chang Hyun Ko, *J. Phys. Chem. B* **2000**, *104,* 11465-11471
